# Facile Detection of Light-Controlled Radical Scavengers from Natural Products Using In Situ UV-LED NMR Spectroscopy

**DOI:** 10.3390/antiox11112206

**Published:** 2022-11-08

**Authors:** InWha Park, Goeun Park, Yoojin Choi, Seung-Woo Jo, Hak Cheol Kwon, Jin-Soo Park, Jin Wook Cha

**Affiliations:** Natural Product Informatics Research Center, KIST Gangneung Institute of Natural Products, Gangneung 25451, Korea

**Keywords:** free radical scavenger, in situ NMR spectroscopy, natural products, photoisomerization

## Abstract

With the recent development of chemical analysis technology, attention has been placed on natural light-sensitive compounds that exhibit photoreactivity to expand the structural diversity of natural product chemistry. Photochemical reactions that proceed via a free radical mechanism could be used to modulate the radical-scavenging ability of natural products as well as involve structural change. As the health benefits of radicals are also presented, there is a need for a controllable radical scavenging method for topical and selective application. In this study, we developed a novel acquisition and processing method to identify light-controlled radical scavengers in plant extracts and evaluate their antioxidant activity under light irradiation based on in situ UV-LED NMR spectroscopy. Using the developed method, licochalcones A and B, in which the *trans* and *cis* isomers undergo reversible photoisomerization, were selectively identified from licorice root extract, and their light-induced free radical scavenging activity was confirmed.

## 1. Introduction

Natural products have served as a repository of useful bioactive substances, including diverse antioxidants, and are still providing new chemical scaffolds [1,2,3,4]. On the other hand, the pharmaceutical industry requiring new pharmaceutical agents faces a new challenge as the re-isolation of known molecules from natural resources is being repeated [5,6]. In this respect, natural compounds that were of little previous interest, such as water-soluble, volatile, and light-sensitive metabolites, are newly emerging with the advancement of analytical instruments [7]. In the process of exploiting bioactive substances and novel secondary metabolites, light is mainly considered to hinder the stability of compounds by occurring photochemical and photocatalytic degradation. In fact, chemicals such as resveratrol, avermectin, and marinomycin are unstable under light irradiation conditions [8,9,10]. However, light plays an essential role in maintaining life by the mediation of bioderived substances, such as chlorophyll and rhodopsin. Additionally, ultraviolet (UV)-absorbing compounds have attracted attention in the pharmaceutical and cosmetic industries by their protecting effects for living organisms from UV damage. Therefore, photoreactive molecules might be used as new bioactive materials. Recently, many light-responsive compounds have been discovered, such as cupriachelin, petrobactin, zerumbone oxide, and dicaffeoylquinic acids, and structural changes due to light irradiation have been elucidated [11,12,13,14]. We have been exploring photoreactive molecules as a way to expand structural diversity originating from nature and discover antioxidant molecules [15,16]. Photochemical reactions are accompanied by electron transfer, which plays a crucial role in generating radicals, such as reactive oxygen species (ROS), in living systems. Therefore, these photoreactive molecules are expected to exhibit antioxidant potential depending on their radical-related characteristics. While natural antioxidants have been discovered to reduce oxidative stress, promoting the development of diseases and the aging process because excess ROS levels cause irreversible damage to cellular structures, a mild level of ROS induces a cellular defense mechanism to prevent cellular damage [17,18]. Moreover, a positive effect of photodynamic therapy using moderately low levels of radicals on human skin has been reported [17,18]. Considering the dual characteristics of radicals, a balance of radicals is more important than entirely eliminating radicals. Accordingly, a method to control the radical scavenging ability of antioxidants is needed. From this point of view, it is possible to discover novel antioxidation modulators by searching for controllable radical scavengers to regulate in vivo ROS. Additionally, as mentioned above, from the close relationship between the photochemical reaction and the inter/intramolecular electron transfer, a photoswitchable compound capable of inducing a photochemical reaction that is reversibly by light irradiation could be the most effective controllable candidate for radical scavenging.

Until now, photoswitchable compounds from various natural sources have mostly been identified by performing pure separations of single molecules and by evaluating their individual photoreactivity [19,20,21,22,23]. However, to more efficiently identify photoswitchable compounds, it is necessary to develop a method to selectively detect photoswitchable compounds and their photoisomerization in various mixtures. Chromatography-based UV spectroscopy and mass spectrometry (MS) are widely used to analyze organic compounds because the measurement process is simple and a high sensitivity is obtained. In principle, however, due to the intrinsic limitations of these methods, such as column retention time and the measurement method, the real-time analysis of photoswitchable compounds under light irradiation by standard chromatographic analysis methods is not possible. Additionally, widely used antioxidant activity evaluation methods, such as 1,1-diphenyl-2-picrylhydrazyl radical (DPPH) and 2,2′-azino-di-(3-ethylbenzthiazoline sulfonic acid) (ABTS) assays [24,25], cannot measure in situ radical scavenging activity under light irradiation conditions. Other spectroscopic methods, such as electron paramagnetic resonance (EPR) spectroscopy, can provide overall antioxidant activity information with high sensitivity, but structural information of specific active compounds contained in the extract cannot be provided [26,27]. On the other hand, nuclear magnetic resonance (NMR) spectroscopy is able to provide atomic-resolution structural information, and due to its noninvasive feature, the method has been widely used in real-time photochemical reaction monitoring of organic compounds [28,29,30,31,32]. However, the existing in situ UV NMR methods have been mainly applied to the analysis of photochemical reactions (photochemistry, photo-CIDNP, etc.) for single compounds [28,29,30,31,32]. Due to the aforementioned shortcomings in sensitivity and the complexity of signal information in the proton NMR spectrum, it is generally impossible to detect the photoswitchable compounds in the chemical mixture and obtain their structure information by any NMR analysis methods. In addition, NMR-based evaluation of the radical scavenging activity of the photoswitchable compounds has also not been reported. In this study, we propose a novel acquisition and processing method to identify light-controlled radical scavengers contained in extracts and to evaluate the antioxidant activity of these scavengers under light irradiation based on in situ UV-light emitting diode (LED) NMR spectroscopy. To this end, we designed a novel NMR processing method tailored to in situ UV-LED NMR acquisition data for the detection of photoswitchable compounds in a mixture and an NMR acquisition method for the evaluation of radical scavenging activity of the photoswitchable compounds using a spin trapping agent.

## 2. Materials and Methods

### 2.1. In Situ UV-LED NMR Spectroscopy

**Figure 1:**
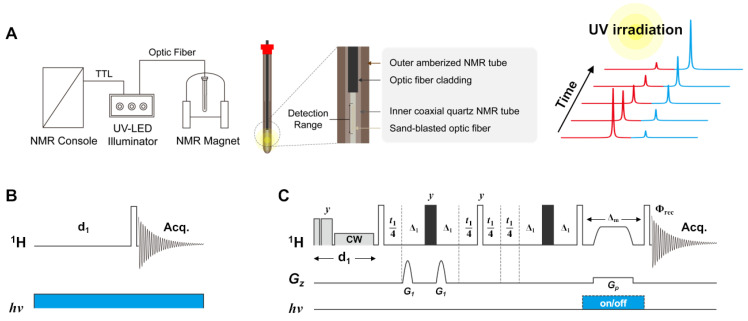
(**A**) Schematic illustration of the in situ UV-LED NMR spectroscopy acquisition system. (**B**,**C**) Pulse sequences for (**B**) 1D in situ UV-LED NMR and (**C**) 2D photo zero-quantum (ZQ) suppression perfect-echo (PE) exchange spectroscopy (2D photo EXSY). The white rectangle, black rectangle, and trapezoid are excitation, inversion, and chirp adiabatic inversion pulses, respectively. The gray rectangles in *d*_1_ delay are for purge pulse and continuous wave for the solvent suppression. The semiellipsoid and the wide white rectangle indicate the gradient pulse. The blue wide rectangle denotes UV irradiation. Delays Δ_1_ are the duration of the sum of the gradient pulse and the following delay, and Δ_m_ indicates the mixing time. In 2D photo EXSY, the duration of UV irradiation time is synchronized with the mixing time. All pulses are of phase *x* unless otherwise indicated. The phase cycling is as follows: Φ_rec_ = *x, −x, −x, x,* and the UV light ignition sequence for the corresponding scan order is on, off, off, and on.

All NMR spectra were measured at 298 K with a 500 MHz Bruker Avance NEO spectrometer equipped with 5 mm CPP BBO probe (Bruker BioSpin, Billerica, MA, USA). For the UV 365 nm light source, fiber-coupled LED, FC5-LED (Prizmatix, Holon, Israel), was employed. For the acquisition of a 1D in situ UV-LED NMR array, the pulse sequence in Figure 1B was used. The *t*_2_ time-domain points were 65536 complex points. For each array, only a single scan with a 90° pulse was carried out. The total delay, which is the sum of acquisition time (Acq.) and delay between each array (*d*_1_), was set to 20 s unless otherwise mentioned. The number of arrays was set according to values indicated in the main text. The total acquisition time of 1D in situ UV-LED NMR array per subfraction sample was about 21 min. For 1D UV-LED acquisition time not mentioned otherwise, it follows the setting in the main text.

For the acquisition of a 2D photo EXSY NMR spectrum, the pulse sequence in Figure 1C was used. The actual time-domain points were 2048 × 64 (*t*_2_ × *t*_1_) complex points. The spectral width was 6579 Hz in both dimensions. The number of scans was 40, and inter scan delay (*d*_1_) was 1 s. The mixing time for UV irradiation was set to 1.5 s. To induce an equilibrium state of photoisomerization, UV 365 nm irradiation was carried out for 10 min before 2D NMR acquisition started. The total acquisition time for 2D photo EXSY NMR per sample was 4 h 12 min.

All NMR solvents used in the NMR acquisition were purchased from Euristop (Saint-Aubin, France). For the acquisition 1D in situ UV-LED NMR array, 5 mg of subfractions of licorice root extract was dissolved in 400 μL of NMR solvent (mixture of 380 μL of dimethyl sulfoxide (DMSO)-*d*_6_ and 20 μL of deuterium oxide). For the acquisition of 2D photo EXSY NMR spectroscopy, each 5 mg of licochacone A (LCA) and licochalcone B (LCB) (Avention, Incheon, Korea) were dissolved in 400 μL of methanol-*d*_4_. In the case of the evaluation of free radical scavenging activity, LCA, LCB, and 4-oxo-TEMPO (Sigma-Aldrich, Saint Louis, MO, USA), according to the concentration indicated in the main text, were dissolved in 400 μL of DMSO-*d*_6_. For the monitoring of L-ascorbic acid (Sigma-Aldrich, Saint Louis, MO, USA) during the photoisomerization of LCB, each 10 mM of the compound was dissolved in DMSO-*d*_6_.

### 2.2. NMR Data Processing

After acquiring the 1D in situ UV-LED NMR arrays, the pseudo-2D NMR data were converted to nmrPipe [33] data format. For the generation of the curvature-edited (CE) spectrum, a home-built processing script according to Equation (3), based on python 3.8 was developed, in which for the input and output of nmrPipe format NMR data, a module for python (nmrglue [34]) was employed.

### 2.3. Preparation of the Subfractions from Licorice Root Extract

The roots of *Glycyrrhiza glabra* were purchased from an herbal garden in Xinjiang, China, in September 2020. A voucher specimen (GU-2009) was deposited at Natural Product Informatics Research Center, Korea Institute of Science and Technology (KIST) Gangneung Institute, Gangneung, Republic of Korea. The roots of *Glycyrrhiza glabra* (0.8 kg) were extracted with ethanol (EtOH) (16 L), and the extract was evaporated in vacuo, and it gave rise to the final 74.0 g of EtOH extract. This extract was suspended in distilled water (1 L) and partitioned sequentially with n-hexane (1 L) and ethyl acetate (EtOAc) (1 L). The EtOAc fraction (6.1 g) was subjected to C18 solid phase extraction (SPE) using a syringe barrel with a pre-packed cartridge, Strata C18 (20 g, Phenomenex, Torrance, CA, USA), and eluted with a stepwise gradient of methanol (MeOH)/water (4/6, 6/4, 8/2; 200 mL for each step) to produce three fractions. For the three fractions, preparative liquid chromatography (LC) was carried out using Waters 1525 Binary HPLC Pump/Full system (Waters, Milford, MA, USA) with a Luna C18 (2) column (150 × 4.6 mm, 5.0 µm, Phenomenex, Torrance, CA, USA). The gradient of the mobile phase was changed from 50/50 to 70/30 [acetonitrile/water] over 90 min with a 12 mL/min of flow rate. As a result, it provided the final eleven subfractions of licorice root extract. Please note that in each preparation step, all the subfractions were concentrated by thoroughly removing the residual organic solvents (ex. acetonitrile).

### 2.4. Tyrosinase Inhibition Activity Assay

Licochalcone A (LCA) and lichocalcone B (LCB) were diluted with Tyrosinase Assay Buffer (25, 100, and 400 μM) from the Tyrosinase Inhibition Screening kit (Sigma-Aldrich, Saint Louis, MO, USA) and placed into a 96-well plate. The plate was exposed to UV light (365 nm) for 30 min to determine whether UV irradiation affected the tyrosinase inhibition activities of LCA and LCB. Samples without UV irradiation were subsequently allocated into the same 96-well plate before performing the tyrosinase inhibition assay. The assay was conducted using a Tyrosinase Inhibitor Screening Kit (Sigma-Aldrich, Saint Louis, MO, USA) following the manufacturer’s protocol. Kojic acid was used as a positive control. The corresponding absorbance values at time points 0 and 60 min were chosen to calculate the relative inhibition rates.

As UV-irradiated LCB caused a distinct increase in the tyrosinase inhibition rate compared to that of nonirradiated LCB, only LCB was used for further experiments. LCB was prepared at lower concentrations (6.25, 25, and 100 μM) since the previous concentrations did not produce tyrosinase inhibition activity in a concentration-dependent manner. After exposing LCB to UV light for 30 min, the samples were left without UV irradiation for different times before the assay was conducted (0, 10, and 20 min). The rest of the assay was performed as described above.

### 2.5. Statistical Analysis

All experiments were performed in triplicate. The data shown are presented as the average of triplicates, and error bars represent the standard deviation (SD). Statistical analysis was conducted using SPSS software. Differences in the tyrosinase inhibition rate among the samples were statistically tested using one-way analysis of variance (ANOVA) and post hoc Tukey’s honestly significant difference (HSD) test. *p* values of <0.05 were considered statistically significant.

## 3. Results and Discussion

### 3.1. Novel NMR Acquisition and Processing Method Tailored to Detect Photoswitchable Compounds

We developed a novel NMR acquisition and processing procedure that enables photoisomerization to be detected in situ and the kinetic and structural modification process of natural compounds from crude extracts to be monitored. For this, an optic fiber-guided UV-LED illumination system was employed for the NMR spectrometer (Figure 1A). UV irradiation during NMR acquisition was controlled by TTL (transistor-transistor logic) communication between the NMR console and the UV-LED illuminator [35].

UV light was then transferred to samples via the optic fiber within a quartz coaxial NMR tube [36], of which the area corresponding to the NMR detection range was sand-blasted for uniform UV irradiation [37]. During NMR acquisition, every single-scan 1D NMR spectrum was recorded as a serial array (*N*) over the UV irradiation time. On the other hand, NMR spectroscopy exhibits relatively low sensitivity and a narrow dynamic range compared to that of other spectroscopic and spectrometric methods. Thus, unlike typical photoisomerization analysis on a single molecule, a small concentration of the photoreactive compounds included in subfraction or crude extract restricts its detection by the simple difference spectroscopic method. Moreover, to perform a quantitative analysis on account of the relatively slow nuclear spin relaxation time ($T_{1}$), the method requires a relatively long inter acquisition delay (>10 s). Therefore, NMR-based real-time acquisition is disadvantaged by low sensitivity and poor time resolution in the detection of photoreactive compounds from mixed samples. To this end, we proposed an alternative NMR data processing method based on the first-order photochemical reaction principle. Assuming that the photoisomerization process obeys the reversible first-order reaction between two chemical species, then the longitudinal magnetization vector $M_{z}$ during an NMR acquisition time, *t*, can be expressed as follows [38]:

(1) $$\frac{d}{dt}M_{z}\left(t\right)=L\left\{M_{z}\left(t\right)-M_{eq}\left(t\right)\right\}+KM_{eq}\left(t\right)$$

where $M_{z}=\left(\begin{array}{c} M_{z}^{1}\\ M_{z}^{2} \end{array}\right),\;L=\left(\begin{array}{cc} -T_{1,1}^{-1}-k_{1} & k_{2}\;\\ k_{1} & -T_{1,2}^{-1}-k_{2} \end{array}\right)\mathrm{and}\;K=\left(\begin{array}{cc} -k_{1} & k_{2}\\ k_{1} & -k_{2} \end{array}\right)$;$\;M_{z}^{j}$, $T_{1,j}$ and $k_{j}$ are the longitudinal magnetization, longitudinal relaxation time, and photoisomerization rate constant of spin *j*, respectively.

If one sets an inter-acquisition delay, $d_{1}$, between every single UV-LED NMR acquisition array, $d_{1}\geq \;5T_{1}$, then the acquired photoisomerization NMR signal every interval *T* has the following form:

(2) $$M_{z}\left(T\right)=\exp\left(KT\right)M_{z}\left(0\right)+\mathrm{random thermal noise}$$

where *T* = $d_{1}$ + Acq.; Acq. = acquisition time per array.

**Figure 2:**
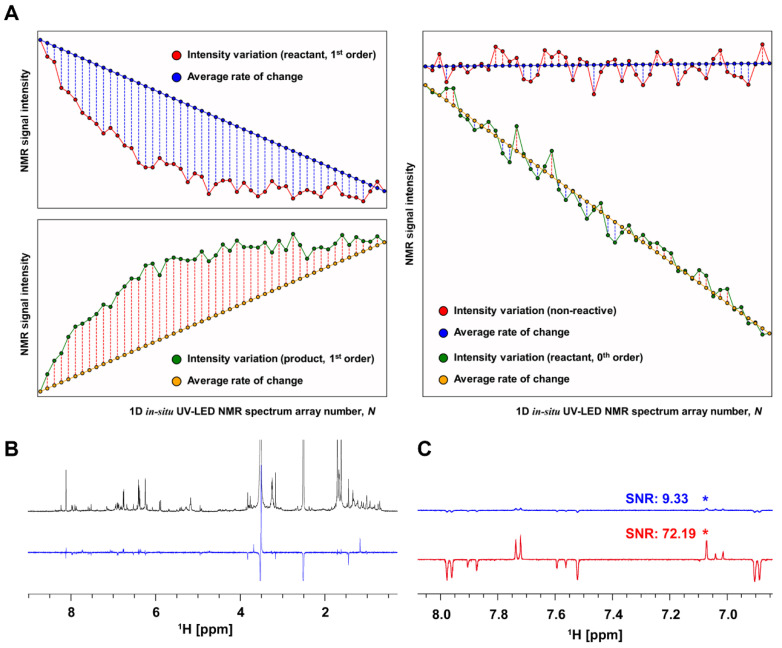
(**A**) Schematic representation of the variation in NMR signal intensity for 1D in situ UV-LED NMR spectral array number according to the type of the photoreaction order (left: 1st order, right: 0th order). (**B**) ^1^H NMR spectrum of a subfraction in the licorice root extract (top) and difference spectrum of the first and last arrays of its 1D in situ UV-LED NMR spectrum (bottom). (**C**) Comparison of each enlarged region of the difference spectrum and the CE spectrum from the subfraction in (**B**); Each SNR value in (**C**) represents a signal-to-noise-ratio of the ^1^H NMR signal under asterisk mark.

For a single pulse NMR acquisition with a pulse angle, *θ*, the NMR signal intensity is proportional to $M_{z}\sin\theta .$ Thus, the NMR signal intensity variation in the photoisomerization compound over UV irradiation time (array number, *N*) is shown as an exponential curve (Figure 2A, left). However, the variation for nonreactive reactants and reactants, which is a zero-order reaction, has a linear form (Figure 2A, right); $d/dtM_{z}\left(t\right)$ is zero (nonreactive) and $d/dtM_{z}\left(t\right)$ is constant (a zero-order reaction). Based on this conclusion, we can define a curvature-edited (CE) NMR spectrum from the in situ UV-LED NMR spectrum arrays in the following equation:

(3) $$C_{j}=\overset{N}{\underset{i=1}{\sum }}S_{i,j}-\left\{\frac{1}{N}\left(S_{N,j}-S_{1,j}\right)\right\}\cdot i-S_{1,j}$$

where *S* is the in situ UV-LED NMR spectrum array matrix, $m_{i}^{T}$ denotes the *i*th array of the 1D in situ UV-LED NMR spectrum and $m_{j}$ denotes the NMR signal amplitude variation along the *j*th column.

The CE spectrum is the result of the accumulation of the differences between the dynamic signal intensity and the average rate of change in signal intensity. As the photoisomerization rate constant $k_{j}$ increases, the area between the average change rate curve and the NMR signal curve tends to increase, whereas as the reaction constant approaches zero, the resulting signal also converges to zero regardless of thermal noise or actual signal intensity differences. Thus, in the CE spectrum, the NMR signals of chemicals that undergo monotonic exponential intensity changes under UV irradiation (365 nm), such as photoisomerization, appear with an enhanced signal-to-noise ratio (SNR) value. Figure 2B shows an example of the ^1^H NMR spectrum of the subfraction of licorice extract (upper, black) and its difference spectrum derived from the in situ UV-LED NMR spectrum array (lower, blue). As stated, due to the small amount of photoreactive compound in the subfraction, the difference spectrum (Figure 2C, upper) shows a weak signal almost indistinguishable from the baseline noise. In contrast, the CE spectrum (Figure 2C, lower) showed the NMR signal of the photoreactive compound with an 8 times higher SNR value than the difference spectrum (72.19 vs. 9.33).

**Figure 3:**
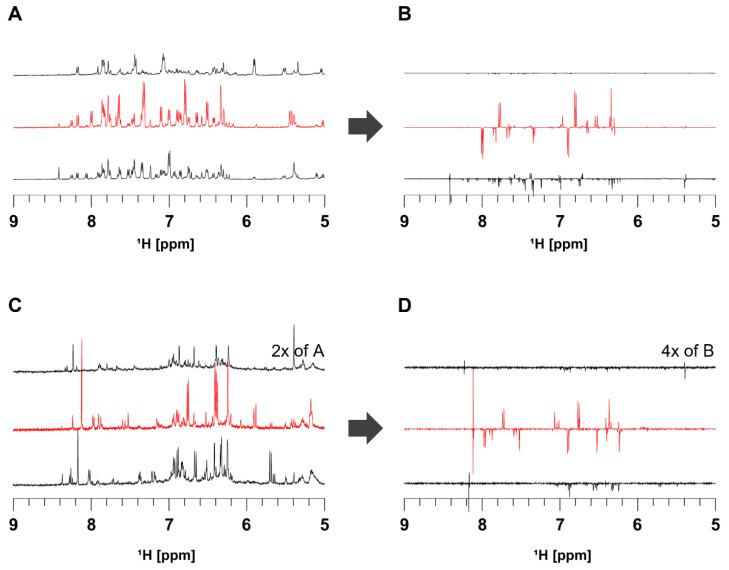
Comparison of stacked ^1^H NMR spectra (left) and corresponding CE spectra (right) for the three successive subfractions in the licorice root extract. (**A**) Expanded ^1^H NMR spectra of subfractions (2, 3, and 4) and (**B**) their corresponding CE spectra. (**C**) Expanded ^1^H NMR spectra of subfractions (8, 9, and 10) and (**D**) their corresponding CE spectra; the number of subfractions is increased from top to bottom of the stacked spectra. (See all ^1^H NMR, difference, and CE spectra of subfractions in Figures S1 and S2).

**Figure 4:**
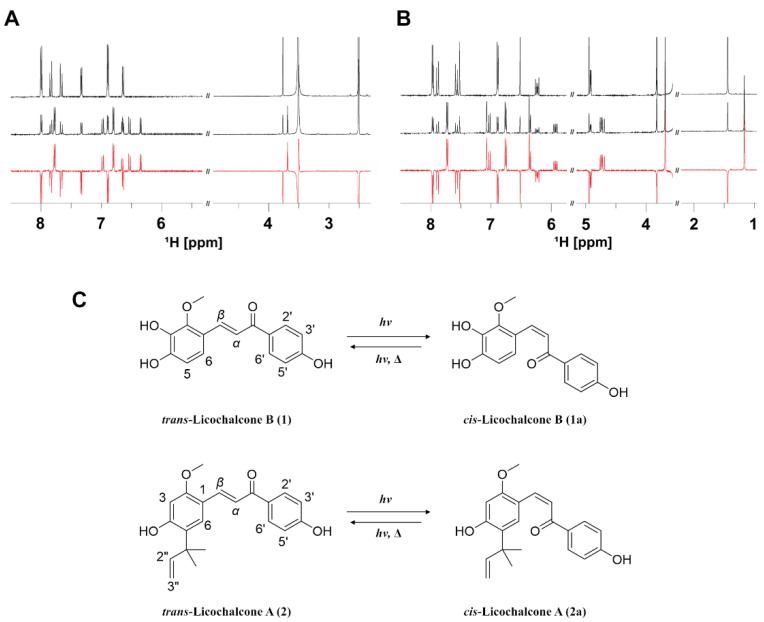
Stacked ^1^H NMR spectra of (**A**) licochalcone B (LCB) and (**B**) licochalcone A (LCA); before (top) and after (middle) UV irradiation of each *trans*-licochalcone A and B, and their difference spectrum (bottom, red). (**C**) Structures of *trans*-licochalcone A and B and their corresponding *cis*-isomers.

Based on the proposed method, to discover photoswitchable natural product compounds from licorice root, we prepared eleven subfractions from the licorice root extract by solid-phase extraction (SPE) followed by column chromatography (CC). Then, we performed in situ UV-LED NMR acquisition (365 nm). For the acquisition of NMR array data, 5 mg of each subfraction was prepared, and the total acquisition time was approximately 21 min per sample. Figure 3 shows successive ^1^H NMR spectra and their corresponding CE spectra. As shown Figure 3B,D, the CE spectrum suggests that two subfractions may contain photoisomerization compounds. According to the definition of Equation (3), a negative NMR signal corresponds to a photoreactive substrate, whereas a positive signal corresponds to a photoreaction product NMR signal. By following the structural information (negative signal) shown in each CE spectrum, we isolated two compounds (1) and (2) from each subfraction and confirmed these compounds by comparison with the CE spectra (Figure 4A,B).

Based on their structures, these compounds were identified as (1) licochalcone B (LCB) and (2) licochalcone A (LCA) by comparison of their NMR data with published values. The corresponding photoreaction isomers of these compounds were also identified as *cis*-LCB (1a) and *cis*-LCA (2a) by the 2D photo NMR spectroscopic analysis described below. (See the detailed structure assignment data for each compound in Supplementary Materials).

### 3.2. Evaluation of Photoisomerization by Kinetic Analysis and 2D Photo NMR Spectroscopy

To determine a type of photoreaction and analyze the structural correlation between substrates and products, we designed a two-dimensional (2D) photo exchange spectroscopy (EXSY) NMR method based on a zero-quantum (ZQ)-filtered perfect-echo (PE) EXSY sequence (Figure 1C). To acquire NMR signals derived from the purely photoreactive process, the following methods were employed for EXSY NMR. In this sequence, the combination of PE sequence and ZQ suppression sequence affords a doubly in-phase cross-peak by preventing anti-phase dispersive NMR signals from being produced, which alleviates signal resolution, thereby hampering the interpretation of NMR signals [39]. Additionally, the nuclear Overhauser effect (NOE) cross-peak due to an intramolecular cross-relaxation during the mixing time was canceled out by the toggled UV irradiation combined with a 180° inversion of a receiver phase for each sequential scan [40]. Consequently, in the doubly Fourier-Transformed (FT) 2D EXSY NMR spectrum, NMR chemical shift information of every ^1^H spin, which belongs to purely photoreaction product and substrate, is provided on each vertical (*F*_1_) and horizontal (*F*_2_) axis. In the 2D EXSY spectrum, NMR signals in a diagonal region (blue) (same chemical shift values at both dimensions) indicate nonreactive ^1^H spins during the UV irradiation progress.

On the other hand, cross-peaks (green) between two different coordinates of chemical shift values are attributed to the photochemical reaction between two ^1^H spins A and B; A $\overset{hv}{\to }$ B. In principle, the direction of the photoreaction can be interpreted through the horizontal section of the 2D EXSY spectrum (from the ^1^H signal at the diagonal peak position to the ^1^H signal at the cross-peak position). Therefore, the structural correlation and the type of photoreaction among them can be analyzed.

**Figure 5:**
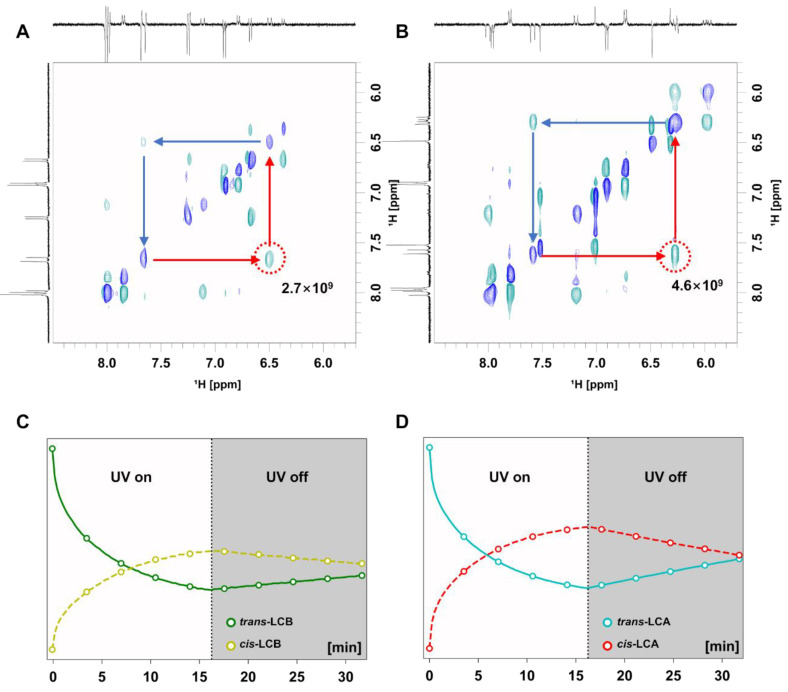
(**A**,**B**) Enlarged 2D photo EXSY NMR spectra of (**A**) LCB and (**B**) LCA; vertical and horizontal traces denote ^1^H NMR spectra of each *trans*-isomer and corresponding difference spectra of the 1D in situ UV-LED spectrum, respectively. Red arrows indicate the direction of *trans*-to-*cis* photoisomerization, and blue arrows indicate the opposite. The values shown in each spectrum represent the integral values of the 2D NMR signal in the red circle. The ratio of the integration of each circled cross-peak per molarity is 1:1.44 [2.7/286.28 vs. 4.6/338.40; LCB vs. LCA]. (**C**,**D**) *Cis–trans* isomerization profile of (**C**) LCB and (**D**) LCA according to UV irradiation conditions. Each point was measured at intervals of 15 s, and total 128 time points were measured; The *y*-axis denotes the NMR intensity of the methoxy group of LCB and the dimethyl group of LCA (in an arbitrary unit). The *x*-axis denotes the monitoring time in minutes.

Figure 5A,B show 2D photo EXSY spectra of LCB and LCA, respectively. In each 2D spectrum, vertical and horizontal traces are the ^1^H NMR spectra and difference spectra of the in situ UV-LED NMR spectrum, respectively. As shown in Figure 5A,B, the cross-peaks (green) appeared as expected. By comparing the vertical and horizontal traces and cross-peaks in the NMR spectrum, structural correlation information between the photoreaction product and substrate could be acquired. Furthermore, symmetry among the cross-peaks could be found based on the diagonal line, which shows a bidirectional photoreaction process during UV irradiation. If the photoreaction is unidirectional, the cross-peak should appear only once between two ^1^H spin coordinates (ex. A $\overset{hv}{\to }$ B); otherwise, it indicates that there a backward photoreaction process occurs during the same UV absorption process. (B $\overset{hv}{\to }$ A). The intensity of the cross-peaks pair can afford a relative photoreaction rate constant between two bidirectional photoreactions. Next, we performed a kinetic analysis of the photoisomerization of licochalcones. Using the same 1D in situ UV-LED NMR method, successive NMR arrays were acquired. To analyze reversible isomerization in dark conditions, which was thermally induced, continuous in situ UV-LED NMR acquisition was performed under UV irradiation conditions (initial 16 min) and dark conditions (late 16 min).

Figure 5C,D show the kinetic curves of the photoisomerization of LCB and LCA, respectively. The signal intensities were collected from the methoxy group of LCB and dimethyl group of LCA and the same position of their corresponding *cis*-isomers. As shown in Figure 5C, the signal intensity of *trans*-LCB (1) showed an exponential decrease, and the signal of *cis*-LCB (1a) showed a complementary increase. In addition, as the UV irradiation time increased, the signal intensity of the two compounds approached the equilibrium state. Afterward, in the dark, the NMR signals underwent a gradual variation in backward intensity, indicating that a thermally induced reversible isomerization had occurred. In addition, according to the increase in UV irradiation time (higher ratio of the *cis* form), the ratio of the two isomers in a state close to equilibrium shows that the rate of photoisomerization of LCB from *trans* to *cis* by UV irradiation is faster than the reverse reaction. The kinetic curve of LCA also showed a similar pattern to that of LCB, but LCA provides a higher ratio of *cis*–*trans* equilibrium ratio than that of LCB (Figure 5D).

### 3.3. In Situ NMR Monitoring of the Radical Scavenging Activity of Licochalcones under UV Irradiation

**Figure 6:**
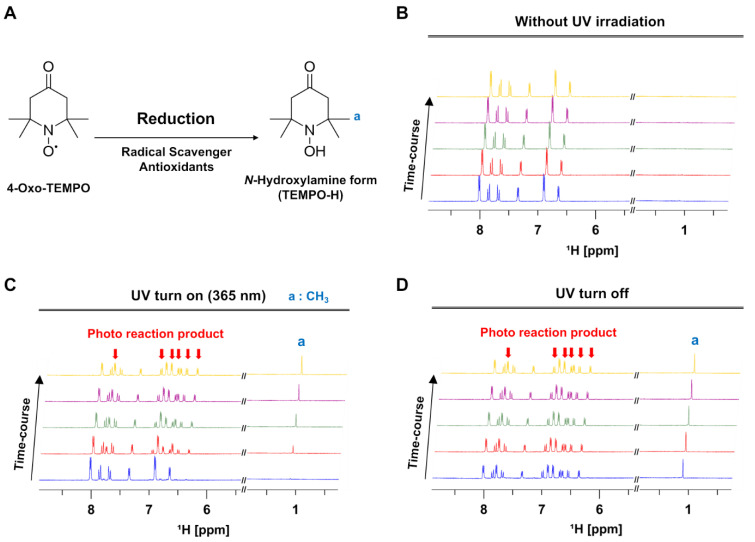
Monitoring the radical scavenging reaction in situ under UV 365 nm irradiation with 1D in situ UV-LED NMR spectroscopy. (**A**) Schematic illustration of the reduction reaction of 4-oxo-TEMPO free radicals by a radical scavenger. (**B**–**D**) Time-course monitoring of the 1D in situ UV-LED NMR signals LCB (25 mM) and TEMPO-H (5 mM). (**B**) Without UV irradiation. (**C**) Upon UV irradiation. (**D**) After the interruption of UV irradiation. Each colored spectrum in (**B**,**C**) shows ^1^H NMR spectrum after 0, 4, 8, 12 and 16 minutes in corresponding measurement condition; From bottom to top, blue, red, green, purple and yellow, respectively.

Next, we evaluated the radical scavenging activity of the isolated photoswitchable compounds LCA and LCB under the photoisomerization process. To measure the radical scavenging effect, 4-oxo-2,2,6,6-tetramethylpiperidin-1-oxyl (4-oxo-TEMPO), which is a stable organic radical compound [41], was employed as a spin probe. In general, a compound containing a free radical such as 4-oxo-TEMPO cannot be observed in the ^1^H NMR spectrum due to a paramagnetic line broadening effect [42]. However, as shown in Figure 6A, 4-oxo-TEMPO can be converted to an *N*-hydroxylamine form of 4-oxo-TEMPO (TEMPO-H) by reduction through interactions with radical scavengers or antioxidant compounds via several reduction mechanisms, single electron transfer (SET) and a hydrogen atom (HAT) [43,44]; thus, the resulting TEMPO-H can be detected as a ^1^H NMR signal. As a result, it is possible to evaluate the radical scavenging activity of the compound of interest indirectly using the NMR signal of the generated TEMPO-H. In the case of 4-oxo-TEMPO, the compound contains four methyl groups; thus, a very strong singlet signal is produced on the NMR spectrum of TEMPO-H (12 protons; *σ*_H_ 1.09 ppm), which is advantageous as the overlap with signals of other compounds is minimized. In addition, since the compound maintains a stable radical form even under UV irradiation conditions (Figure S3), it can be effectively used to evaluate the antioxidant activity that appears in photoreaction processes, such as photoisomerization.

Radical scavenging activity under the photoisomerization process was monitored on a mixed sample of LCB (25 mM) and 4-oxo-TEMPO (5 mM) based on the same in situ UV-LED NMR method, and 1D NMR arrays under the three conditions (without UV, UV turned on and off) were each successively acquired for approximately 16 min. As shown in Figure 6B, when UV irradiation was omitted from the initial measurement condition, there were no changes in the NMR arrays, and the TEMPO-H signal was not detected at all. On the other hand, under UV irradiation (Figure 6C), NMR signal changes were observed for each LCB isomer due to photoisomerization, as in the previous results; at the same time, the TEMPO-H signal also increased according to the UV irradiation time. Upon the interruption of UV irradiation (Figure 6D), the increase in the TEMPO-H signal was stopped, and the signal change of *cis*–*trans*-LCB by thermal reverse isomerization appeared. The same experiment was also performed on LCA, and the signal pattern of TEMPO-H generation showed similar results to that of LCB. However, the TEMPO-H signal was approximately about six times weaker than that of LCB (Figure S4).

### 3.4. Correlation between the Radical Scavenging Activity and Photoisomerization of Licochalcones

**Figure 7:**
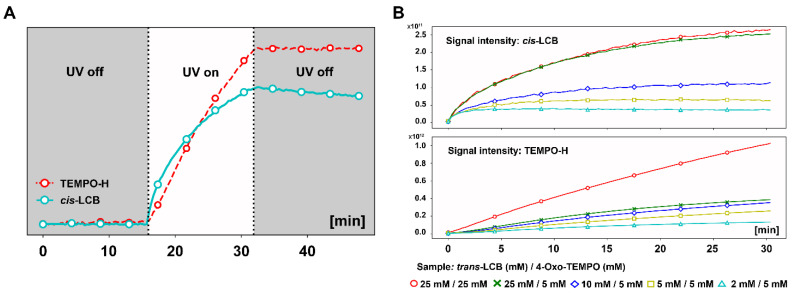
(**A**) NMR intensity profile of *cis*-LCB and TEMPO-H according to UV 365 nm irradiation from Figure 6. Total of 144 time points were measured. (**B**) NMR signal intensity profile of *cis*-LCB (upper) and TEMPO-H (lower) according to concentration changes under UV 365 nm irradiation. Total of 92 time points were measured. The *y*-axis denotes the signal intensity of *cis*-LCB and TEMPO-H according to the concentration of *trans*-LCB and 4-oxo-TEMPO, respectively (in arbitrary units). The *x*-axis denotes UV irradiation time in minutes.

**Scheme 1:**
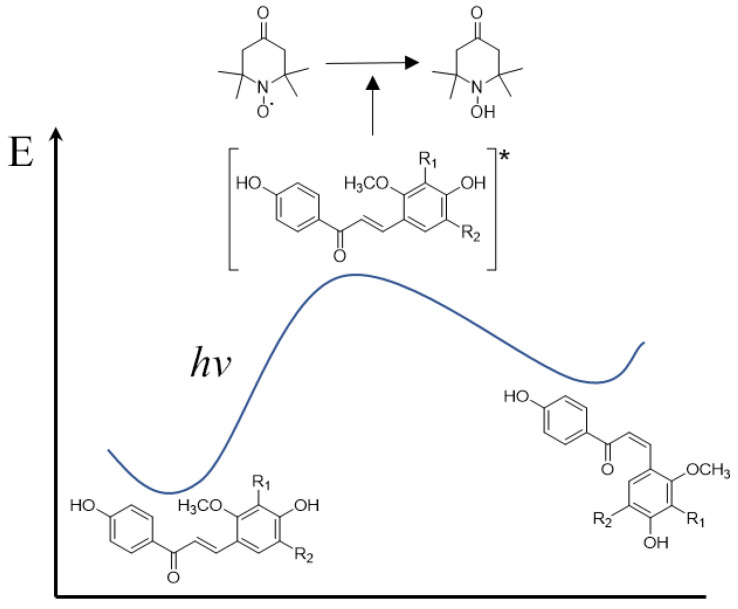
Proposed mechanism for the radical scavenging effect of licochalcones under the photoisomerization process.

To analyze the correlation between the radical scavenging reaction of 4-oxo-TEMPO and the photoisomerization process of licochalcones, we monitored the rate of generation and amount of each product (*cis*-LCB and TEMPO-H) according to the amount of each *trans*-LCB and 4-oxo-TEMPO in concert with an increased UV irradiation time. In Figure 7B, *cis*-LCB was generated in proportion to the initial *trans*-LCB concentration regardless of the amount of 4-oxo-TEMPO. On the other hand, the rate of generation and amount of TEMPO-H were changed according to the concentration change of *cis*-LCB and 4-oxo-TEMPO. Interestingly, in the comparison of two conditions ([25 mM/5 mM] and [10 mM/5 mM]; [Conc. of *trans*-LCB/4-oxo-TEMPO]), a difference in the initial production rate of TEMPO-H was observed, but the final production amount was almost similar. This could be due to the effect of a greater amount of LCB than 4-oxo-TEMPO. Consequently, based on the monitoring results of TEMPO-H generation according to UV irradiation and substrate concentration, we proposed a light-induced radical scavenging activity mechanism via the photoisomerization process (Scheme 1). As revealed in many previous research results [45,46], the ground-state photoswitchable compound is converted into a corresponding isomer via an excited state (ES) intermediate through energy absorption by light irradiation.

Additionally, as mentioned above, the reduction product of 4-oxo-TEMPO was produced only upon UV irradiation regardless of the presence of *cis*–*trans* compounds suggesting no direct reaction between *cis*–*trans* compounds and 4-oxo-TEMPO, and it was confirmed that the reaction rate was proportional to the concentration of LCA/LCB and free-radical compounds. In addition, to determine whether ROS or singlet oxygen was generated by the photosensitizer action of licochalcones, structural changes in ascorbic acid, a well-known antioxidant, were monitored during the photoisomerization of LCB. As a result, no chemical change in ascorbic acid was observed during the photoisomerization of LCB according to UV irradiation (Figure S5).

Hence, we concluded that the radical scavenging reaction occurs by the interaction between the photoisomerization ES intermediate of licochalcones and free radicals. Furthermore, since the volume of the cross-peak per molarity in the 2D photo EXSY spectrum is closely related to the rate of each *cis* ↔ *trans* isomerization reaction; for example, as shown in Figure 5A,B, the integration ratio of cross-peaks between H-α signals of each isomer showed a ratio of 1 (LCB) to 1.44 (LCA), it can be inferred that LCA undergoes faster photoisomerization than that of LCB. As a result, a difference in the duration of ES intermediate was observed in the photoisomerization process between LCA and LCB (longer duration in LCB), which is also in good agreement with the results of different 4-oxo-TEMPO radical scavenging activities of LCA and LCB (higher TEMPO-H signal intensity at LCB).

### 3.5. Tyrosinase Inhibition of Licochalcones by UV Irradiation

After human skin is exposed to ultraviolet A (UVA), ROS production is induced, and excessive melanin is accumulated in skin cells [47]. In the melanogenesis signaling pathway, ROS synthesized by UVA is known to trigger the production of α-melanocyte stimulation hormone (α-MSH), mediating the accumulation of melanin in melanosomes [48]. Thus, when the antioxidant material exhibits a whitening effect together, the usefulness of the material might be strengthened. Melanogenesis inhibitors are mainly screened through evaluation against tyrosinase, which catalyzes two rate-limiting steps to synthesize melanin through the hydroxylation of L-tyrosine to 3,4-dihydroxy-L-phenylalanine (L-DOPA) and the oxidation of L-DOPA to DOPA quinine [49].

**Figure 8:**
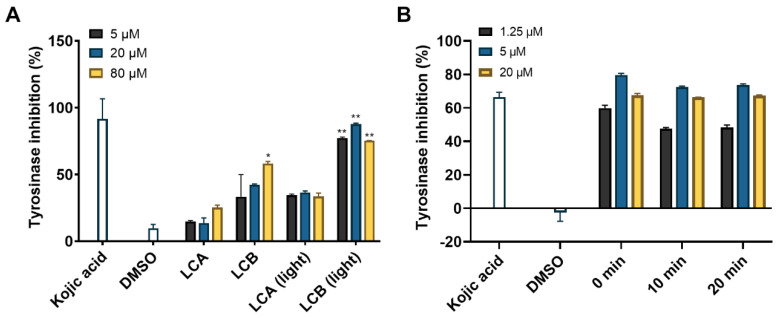
(**A**) Relative tyrosinase inhibition rate of UV-irradiated and nonirradiated LCA and LCB. (**B**) Relative tyrosinase inhibition rate of LCB at different time points after termination of UV irradiation. The final concentrations of the samples are presented. Kojic acid (150 μM) was used as a positive control. Data are shown as the mean ± SD (*n* = 3). Significant differences compared with one another were analyzed by one-way ANOVA, post hoc tests, and Tukey’s HSD test (* *p* < 0.05, ** *p* < 0.01).

To further utilize licochalcones with the light-induced radical scavenging activity, their inhibitory effect according to UVA irradiation was evaluated against tyrosinase. When three different concentrations of LCA and LCB (final concentrations: 5, 20, and 80 μM) were exposed to 365 nm UV light, the relative tyrosinase inhibition rates of both compounds seemed to increase when compared to that of nonirradiated samples (Figure 8A). In the case of LCB, the increase in the inhibition rate after UV irradiation was found to be statistically significant (*p* < 0.01). This result confirms that UV irradiation of licochalones induces an increase in the tyrosinase inhibitory effect as well as the radical scavenging ability, so it might be effective in suppressing melanogenesis.

The relative tyrosinase inhibition rates of LCB at different times (0, 10, and 20 min) since UV irradiation was terminated are presented in Figure 8B. There was a slight decrease in the relative tyrosinase inhibition rate when tyrosinase was incubated for a longer time after UV irradiation at concentrations of 5 and 1.25 μM. However, the decrease in the inhibition rate was negligible, and the tyrosinase inhibition rate of LCB at a concentration of 20 μM showed almost no changes among the different time points. As the inhibition of enzyme activity is maintained immediately after UV irradiation is finished, LCB is expected to be useful as a whitening material for licochalcones and licorice in a UV-exposed environment.

## 4. Conclusions

In this paper, we proposed a simple method for the detection and evaluation of light-controlled radical scavengers from natural products based on in situ UV-LED NMR spectroscopy. The feasibility of this method was demonstrated for the licorice root extract; as a result, two photoswitchable chalcones, licochalcones A and B, were identified. We evaluated their light-induced free radical scavenging activity and suggested a plausible free radical scavenging mechanism through the photoisomerization of licochalcones. Based on these results, we were able to propose novel light-controlled antioxidants. Our proposed method for the effective detection of novel radical scavengers should be effectively utilized to find or re-evaluate antioxidants from natural sources, including many known natural product compounds.

## Data Availability

Not applicable.

## Supplementary Materials

The following supporting information can be downloaded at: https://www.mdpi.com/article/10.3390/antiox11112206/s1.
