# Supplementary material for: Facile Detection of Light-Controlled Radical Scavengers from Natural Products Using In Situ UV-LED NMR Spectroscopy
Source: Antioxidants (Basel). 2022 Nov 8;11(11):2206. doi: 10.3390/antiox11112206 (PMC9687055; doi:10.3390/antiox11112206)
Supplement: Supplementary file 1 [file antioxidants-11-02206-s001.zip › antioxidants-1977159-supplementary.pdf]

antioxidants

MDPI

Supplementary Materials

Identification and Structure Elucidation of Four Photoswitchable Compounds

The ^{1}H NMR data of compound 1 under the condition of blocking light exhibited two benzene rings, one *α*, *β*-double bond with the coupling constant of 15.7 Hz, which indicated the *E*-configuration, one vinyl, one methoxy, and two methyl groups. Compound 1 was identified as *trans*-licochalcone B (*trans*-LCB) by the comparison of their spectroscopic data with published values [1]. Upon UV irradiation at 365 nm, *trans*-LCB was transformed into its *cis*-isomer (1a). Negative peaks appearing in the difference spectrum of the before and after light irradiation were assigned as *cis*-licochalcone B (*cis*-LCB). The *cis*-configuration of 1a was determined by coupling constants $J_{H\text{-}\alpha/H\text{-}\beta}$ = 12.6 Hz. The configuration of photo-isomer 1a was supported by equilibrium spots present in the photo EXSY spectrum. The 2D photo EXSY cross-peaks of the *trans*-double bond at 7.98/7.64 ppm and the *cis* double bond at 7.06/6.44 ppm.

The ^{1}H NMR spectrum of compound 2 without light exposure was similar to those of 1. The configuration of the double bond in 2 was found to be *trans*, as deduced from the coupling constants $J_{H\text{-}\alpha/H\text{-}\beta}$ = 15.6 Hz. The structure of 2 was established as *trans*-licochalcone A by the comparison of their spectroscopic data with published values [2]. The UV irradiation of *trans*-2 resulted in photo-isomer (2a). In the difference data of the before and after light irradiation, negative signals were identified as *cis*-licochalcone A. The coupling constant $J_{H\text{-}\alpha/H\text{-}\beta}$ = 12.7 Hz confirmed *cis*-configuration at the *α*, *β*-double bond. The 2D photo EXSY NMR spectrum of 2 with UV revealed cross-peaks between the *trans*-double bond at 7.98/7.56 of 2 and the *cis*-double bond at 7.17/6.25 of 2a.

trans-Licochalcone B (1): ^{1}H NMR (500 MHz, methanol-*d*4) δ_H 7.98 (3H, overlapped, H-2′, 6′, β), 7.64 (1H, d, *J* = 15.7 Hz, H-*α*), 7.23 (1H, d, *J* = 8.7 Hz, H-6), 6.89 (2H, d, *J* = 8.7 Hz, H-3′, 5′), 6.65 (1H, d, *J* = 8.7 Hz, H-5), 3.85 (3H, s, -OCH_3); ^{1}H NMR (500 MHz, DMSO-*d*6) δ_H 8.00 (2H, d, *J* = 8.6 Hz, H-2′, 6′), 7.84 (1H, d, *J* = 15.7 Hz, H-β), 7.67 (1H, d, *J* = 15.7 Hz, H-*α*), 7.33 (1H, d, *J* = 8.4 Hz, H-6), 6.88 (2H, d, *J* = 8.6 Hz, H-3′, 5′), 6.63 (1H, d, *J* = 8.4 Hz, H-5), 3.77 (3H, s, -OCH_3)

cis-Licochalcone B (1a): ^{1}H NMR (500 MHz, methanol-*d*4) δ_H 7.79 (2H, d, *J* = 8.6 Hz, H-2′, 6′), 7.06 (1H, d, *J* = 12.6 Hz, H-β), 6.73 (2H, d, *J* = 8.6 Hz, H-3′, 5′), 6.62 (1H, d, *J* = 8.5 Hz, H-6), 6.44 (1H, d, *J* = 12.6 Hz, H-*α*), 6.31 (1H, d, *J* = 8.5 Hz, H-5); ^{1}H NMR (500 MHz, DMSO-*d*6) δ_H 7.77 (2H, d, *J* = 8.6 Hz, H-2′, 6′), 6.97 (1H, d, *J* = 12.9 Hz, H-β), 6.79 (2H, d, *J* = 8.6 Hz, H-3′, 5′), 6.67 (1H, d, *J* = 8.4 Hz, H-6), 6.53 (1H, d, *J* = 12.9 Hz, H-α), 6.34 (1H, d, *J* = 8.4 Hz, H-5), 3.69 (3H, s, -OCH_3)

trans-Licochalcone A (2): ^{1}H NMR (500 MHz, methanol-*d*4) δ_H 7.98 (1H, d, *J* = 15.6 Hz, H-β), 7.94 (2H, d, *J* = 8.4 Hz, H-2′, 6′), 7.56 (1H, d, *J* = 15.6 Hz, H-*α*), 7.50 (1H, s, H-6), 6.89 (2H, d, *J* = 8.4 Hz, H-3′, 5′), 6.46 (1H, s, H-3), 6.25 (1H, dd, *J* = 17.7, 10.5 Hz, H-2″), 5.00 (1H, d, *J* = 4.7 Hz, H_2-3″a), 4.97 (1H, s, H_2-3″b), 3.88 (3H, s, OCH_3), 1.48 (6H, s, -CH_3 × 2); ^{1}H NMR (500 MHz, DMSO-*d*6) δ_H 7.97 (2H, d, *J* = 8.1 Hz, H-2′, 6′), 7.89 (1H, d, *J* = 15.5 Hz, H-β), 7.58 (1H, d, *J* = 15.5 Hz, H-*α*), 7.52 (1H, s, H-6), 6.88 (2H, d, *J* = 8.1 Hz, H-3′, 5′), 6.52 (1H, s, H-3), 6.25 (1H, dd, *J* = 17.5, 10.5 Hz, H-2″), 4.94 (2H, overlapped, H_2-3″a, 3″b), 3.82 (3H, s, -OCH_3), 1.45 (6H, s, -CH_3 × 2) *cis*-Licochalcone A (2a): ^{1}H NMR (500 MHz, methanol-*d*4) δ_H 7.78 (2H, d, *J* = 8.3 Hz, H-2′, 6′), 7.17 (1H, d, *J* = 12.6 Hz, H-β), 6.99 (1H, s, H-6), 6.71 (2H, d, *J* = 8.3 Hz, H-3′, 5′), 6.30 (1H, s, H-3), 6.25 (1H, d, *J* = 12.6 Hz, H-*α*), 5.95 (1H, dd, *J* = 17.4, 10.5 Hz, H-2″), 4.76 (2H, m, H_2-3″), 3.73 (3H, s, -OCH_3), 1.20 (6H, s, -CH_3 × 2); ^{1}H NMR (500 MHz, DMSO-*d*6) δ_H 7.73 (2H, d, *J* = 8.2 Hz, H-2′, 6′), 7.10 (1H, s, H-6), 7.02 (1H, d, *J* = 12.8 Hz, H-β), 6.75 (2H, d, *J* = 8.2 Hz, H-3′, 5′), 6.36 (2H, overlapped, H-3, H-*α*), 5.95 (1H, dd, *J* = 17.4, 10.6 Hz, H-2″), 4.73 (2H, overlapped, H_2-3″a, 3″b), 3.68 (3H, s, -OCH_3), 1.17 (6H, s, -CH_3 × 2)

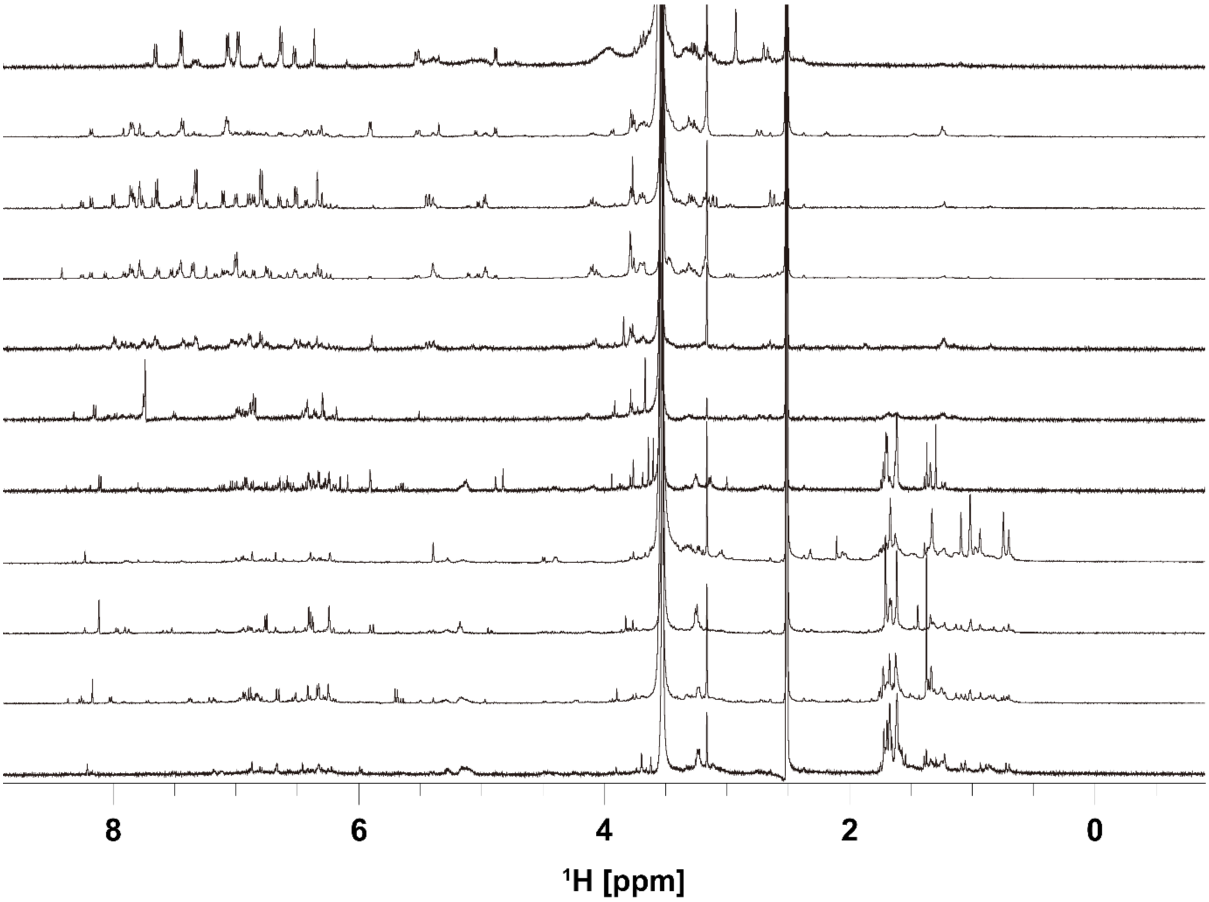

Figure S1. Stacked ^{1}H NMR spectra of eleven subfractions from licorice root extract. The number of each subfraction increases in order from top to bottom of the spectrum.

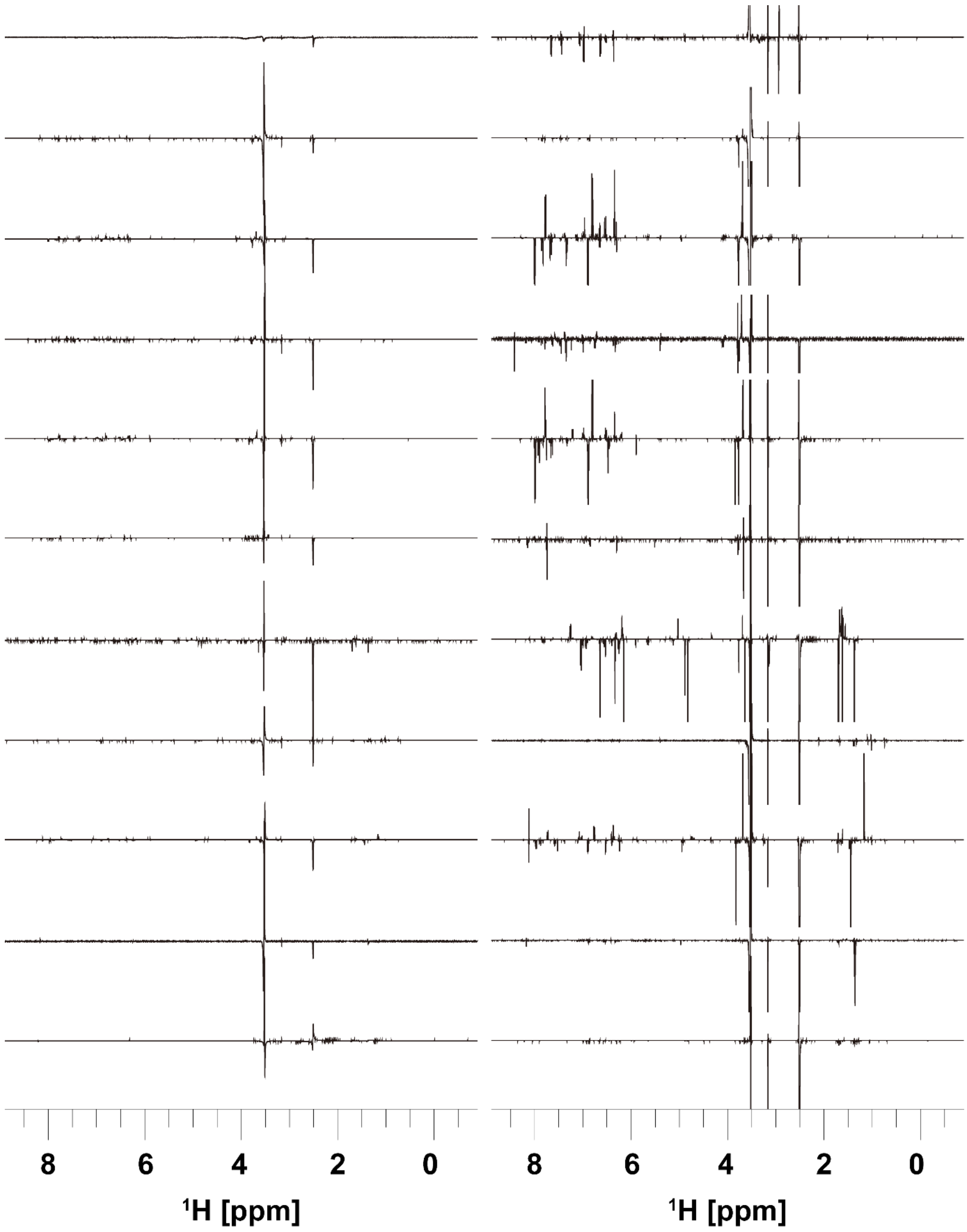

Figure S2. Comparison of the difference spectra (left) and the CE spectra (right) of 1D in situ UVLED spectra (365 nm) of subfractions from licorice root extract; The number of each subfraction increases in order from top to bottom of the spectrum. Note that in fifth CE spectrum, it also showed NMR signals indicating photoreactive compounds, and it turned out that those signals were attributed to LCB.

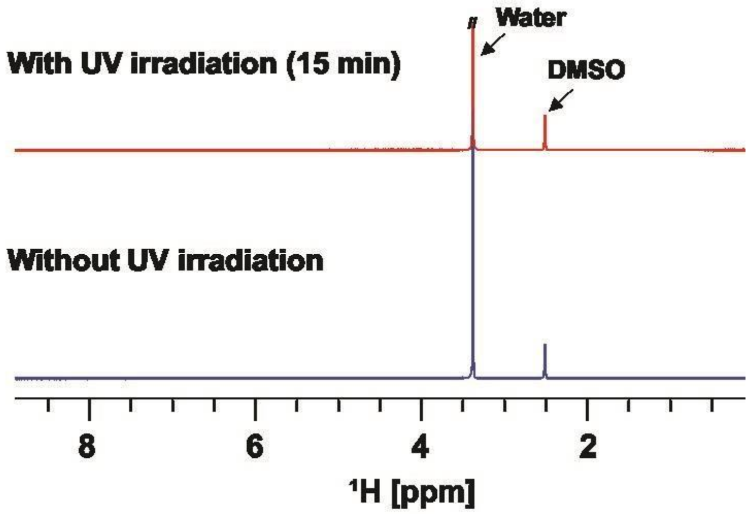

Figure S3. Stacked spectra of ^{1}H NMR spectrum of 4-oxo-TEMPO after UV irradiation (365 nm) (upper) and before UV irradiation (lower). Both NMR spectra before and after UV irradiation showed no NMR signal.

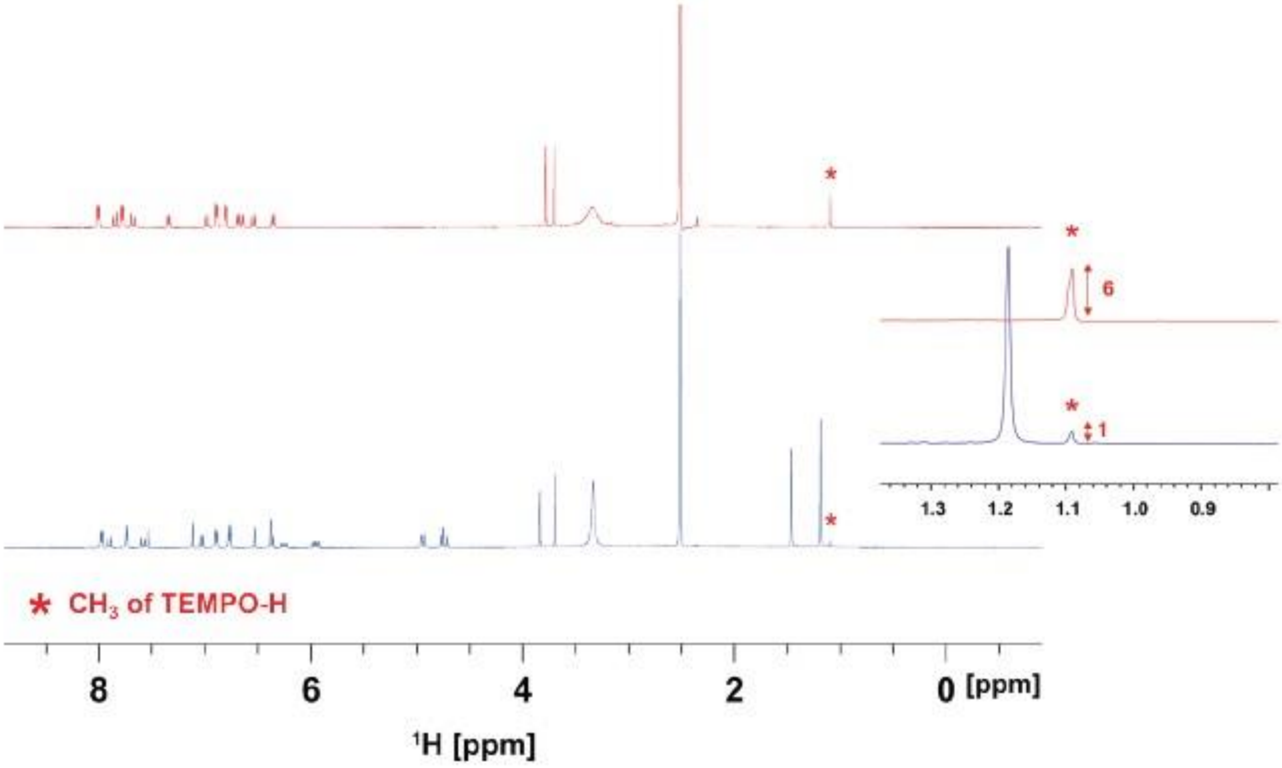

Figure S4. Comparison of NMR signal intensity of CH_3 in TEMPO-H after UV 365 nm irradiation (about 15 min). The mixture of 4-oxo-TEMPO (5 mM) with 25 mM of LCA (lower) and 25 mM of LCB (upper) in DMSO-*d*6, respectively. Inset is an enlarged region of CH_3 signal in TEMPO-H. The ratio of the signal intensity of CH_3 was 6:1 [LCB:LCA]. The asterisk marks on the ^{1}H NMR spectra indicate the methyl signal of the TEMPO-H.

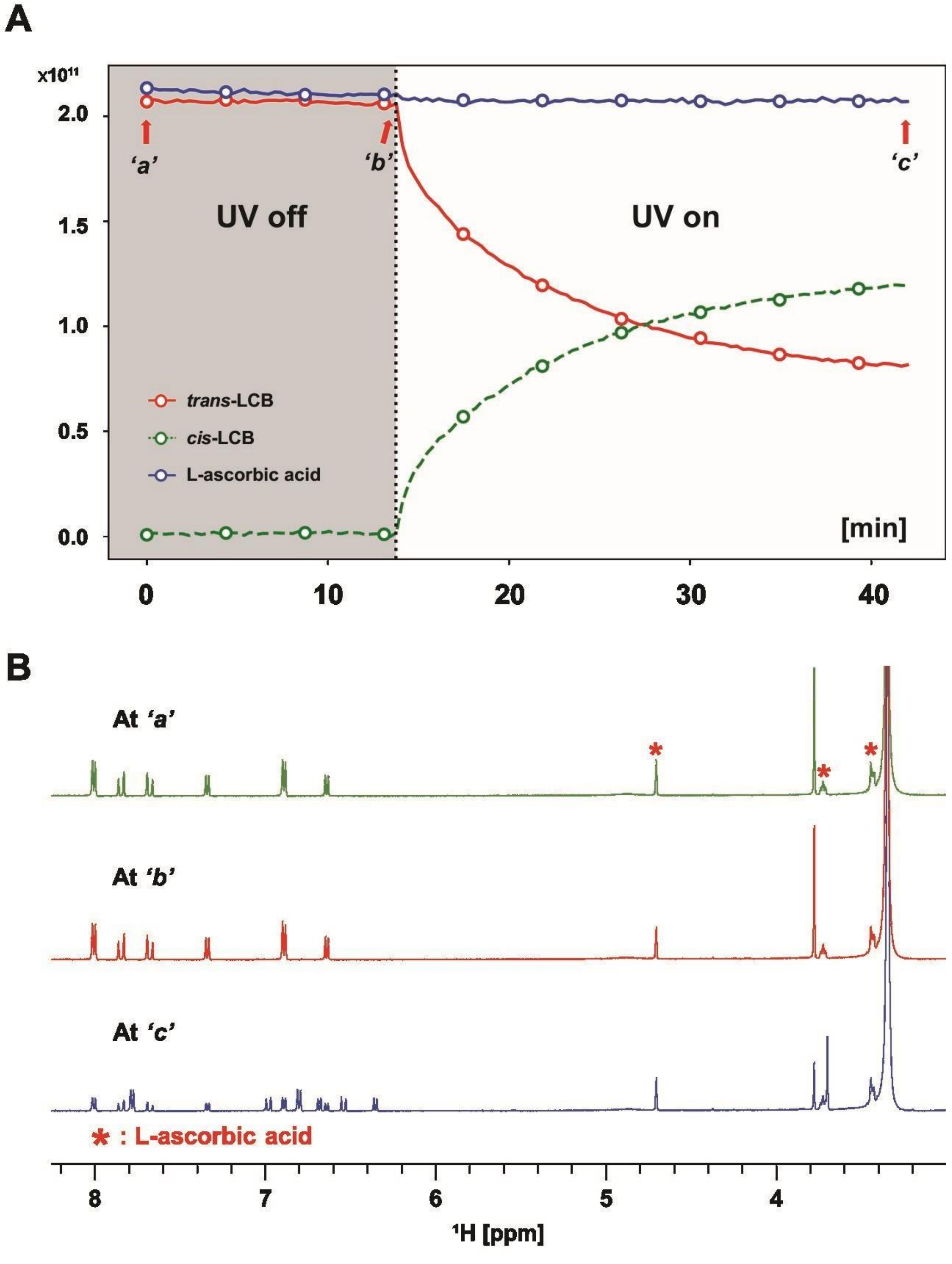

Figure S5. Monitoring of L-ascorbic acid during photoisomerization of LCB. (**A**) NMR signal profile of LCB and L-ascorbic acid according to UV 365 nm irradiation condition. (**B**) Stacked ^{1}H NMR spectra at each array point indicated in (**A**). As shown in lower (blue) ^{1}H NMR spectrum in (**B**), photoisomerization of LCB by UV irradiation was observed, but no change in any NMR signal was observed due to the reaction of L-ascorbic acid with ROS. The asterisk marks on the 1H NMR spectrum indicate ^{1}H NMR signals of the L-ascorbic acid.

References

1. Xiao, Y.; Han, F.; Kim, M.J.; Lee, K.Y.; Lee, I.S. Microbial transformation of broussochalcones A and B by *Aspergillus niger*. *J. Nat. Prod*. **2021**, *84*, 601–607.
2. Wang, J.; Zhang, Y.S.; Thakur, K.; Hussaina, S.S.; Zhang, J.G.; Xiao, G.R.; Wei, Z.J. Licochalcone A from licorice root, an inhibitor of human hepatoma cell growth via induction of cell apoptosis and cell cycle arrest. *Food Chem. Toxicol*. **2018**, *120*, 407–417.
